# Drug-drug interaction signals between carbonic anhydrase inhibitors and vitamin D preparations in urinary tract stones: disproportionality analysis evaluation from Japanese spontaneous reports of adverse events

**DOI:** 10.1186/s40780-026-00574-2

**Published:** 2026-04-27

**Authors:** Teruhisa Kinoshita, Yuki Kondo, Yuka Sakazaki, Norio Takimoto, Yoichi Ishitsuka

**Affiliations:** 1 Department of Clinical Chemistry and Informatics, Graduate School of Pharmaceutical Sciences, Kumamoto University, 5-1 Oehonmachi, Chuo-ku, Kumamoto, 862-0973 Japan; 2 Department of Pharmacy, Kariya Toyota General Hospital, Kariya-city, Aichi Prefecture Japan

**Keywords:** Carbonic anhydrase inhibitors, Vitamin D, Urinary tract stones, Drug-drug interactions, Japanese Adverse Drug Event Report

## Abstract

**Background:**

Carbonic anhydrase inhibitors used as antiepileptic drugs promote calcium phosphate stones. Patients with epilepsy often receive vitamin D concurrently, which may increase urinary calcium and stone formation. We analyzed drug-drug interactions to determine if vitamin D contribute to renal and urinary tract stone disease associated with carbonic anhydrase inhibitors.

**Methods:**

The Japanese Adverse Drug Event Report (JADER) was used to detect signals of the additive effects of vitamin D on carbonic anhydrase inhibitor-associated renal and urinary tract stone disease. Disproportionality analysis and Ω shrinkage measure were used to detect renal and urinary tract stone disease signals when carbonic anhydrase inhibitors were used with vitamin D.

**Results:**

We found 61 cases of carbonic anhydrase inhibitor-related renal and urinary tract stone disease. When carbonic anhydrase inhibitors were combined with vitamin D preparations, a significant positive signal was observed for renal and urinary tract stone disease exclusively (aROR 59.85, 95% CI 29.04–123.36, *p* < 0.001), compared to that with carbonic anhydrase inhibitor (aROR 12.99; 95% CI 9.69–17.41; *p* < 0.001) or vitamin D alone (aROR 8.68, 95% CI 6.98–10.79, *p* < 0.001). A significant positive signal was also detected for the Ω shrinkage measure (Ω025 = 0.429).

**Conclusion:**

Vitamin D preparations may adversely affect renal and urinary tract stone disease when used with carbonic anhydrase inhibitors, and the hypothesis should be further investigated in future research.

**Supplementary Information:**

The online version contains supplementary material available at 10.1186/s40780-026-00574-2.

## Background

Topiramate and zonisamide, among others used as antiepileptic drugs, exert carbonic anhydrase inhibitory effects. These drugs are associated with an increased risk of developing urinary stones because of carbonic anhydrase inhibition [1].

Several mechanisms are believed to contribute to stone formation. The first mechanism is increased urinary excretion of calcium and phosphate, which may increase the risk of calcium phosphate stone formation [2, 3]. The second mechanism is urinary alkalinization. Carbonic anhydrase inhibitors alkalize urine by promoting bicarbonate excretion. Reportedly, when the urine pH exceeds 6, the risk of calcium phosphate stone formation increases [4]. The third mechanism is decreased urinary citrate excretion. Carbonic anhydrase inhibitors have been reported to reduce urinary citrate excretion [5, 6], which plays a key role in preventing stone formation by nucleation and crystal growth [7]. Patients with epilepsy are known to have a higher risk of fractures [8, 9] and are prone to vitamin D deficiency [10]. Vitamin D preparations such as eldecalcitol are used to treat osteoporosis and are effective in reducing vertebral fractures [11]. However, high-dose vitamin D intake has been shown to increase urinary calcium excretion [12, 13], raising concerns about an increased risk of calcium phosphate stones. However, to our knowledge, no reports to date have shown an increased risk of developing urinary stones when these medications are used in combination.

Therefore, we hypothesized that the combined use of carbonic anhydrase inhibitors and vitamin D preparations may increase the risk of kidney and urinary stones by increasing urinary calcium concentrations.

As data on the concomitant use of carbonic anhydrase inhibitors and Vitamin D are limited, disproportionality analysis using a large Individual Case Safety Report (ICSR) database may serve as a useful approach for hypothesis generation.

The purpose of this study was to conduct a signal detection analysis of drug-drug interactions using the Japanese Adverse Drug Event Report (JADER) to generate a hypothesis regarding the interaction between vitamin D preparations and carbonic anhydrase inhibitors in renal and urinary tract stone disease.

Of note, this study aims to detect signals and generate hypotheses. It does not consider causal inference to validate causal relationships.

## Materials and methods

### Study population and data collection

This study follows the ‘REporting of A Disproportionality analysis for drUg Safety signal detection using individual case safety reports in PharmacoVigilance’ (READUS-PV) statement.

As our analysis was based on a publicly available side-effect database, informed consent was not required. Data were obtained from JADER, which collects reports of adverse events submitted to the Pharmaceuticals and Medical Devices Agency and those from public sources. Data from April 2004 to September 2025 were downloaded from the Pharmaceuticals and Medical Devices Agency website on October 8, 2025. JADER consists of four datasets: “DEMO” (demographic information), “DRUG” (medication administration information), “REAC” (adverse event information), and “HIST” (comorbidity information). Data were cleaned to remove duplicates and incomplete records. Specifically, reports with missing information on sex or age were excluded from the analysis (complete-case analysis), as these variables are required as covariates in the multivariate logistic regression. Age information was stored in 10-year intervals in the “DEMO” dataset. The DRUG table contains several categories, including suspected drugs, concomitant drugs, and drug interactions. In this study, drugs were extracted from all of these categories when identifying carbonic anhydrase inhibitors and vitamin D.

### Adverse event detection

In this study, we used the Japanese version of the Medical Dictionary of Research (MedDRA), MedDRA/J ver. 28.1J. Renal or urinary stone events were identified using the appropriate Preferred Term (PT) codes (Table 1). PTs were selected by comprehensively searching for terms related to ‘nephrolithiasis’ and ‘urolithiasis’ in MedDRA and identifying those that were clinically relevant to the objective of this study. In accordance with the MedDRA Term Selection: Points to Consider document (Section 3.13, Medical and Surgical Procedures), we prioritized diagnostic PTs over procedural terms because the latter are generally considered inappropriate for representing adverse events when a definitive diagnosis can be identified. Information including sex, age, year of reporting, and renal and urinary stone events was extracted.

**Table 1:** Preferred term (PT) code used in the analysis

PT code	
10006987	Calculus bladder
10007019	Calculus prostatic
10007026	Calculus urethral
10007027	Calculus urinary
10029148	Nephrolithiasis
10052479	Seminal vesicular calculus
10041900	Stag horn calculus
10077989	Ureterolithiasis

The medications studied included carbonic anhydrase inhibitors (acetazolamide, zonisamide, topiramate, sulthiame) and vitamin D preparations (alfacalcidol, calcitriol, tacalcitol, falecalcitriol, eldecalcitol).

Carbonic anhydrase inhibitors are also used as eye drops, but as their systemic absorption is very small, they were excluded from the study [14]. Further, topical vitamin D preparations are known to induce a certain increase in blood vitamin D concentrations, but these were excluded because a detailed reading of the daily application amount could not be obtained from the database.

### Statistical analysis

To detect signals of renal stones and urolithiasis associated with carbonic anhydrase inhibitors, disproportionality analysis was performed using 2 × 2 contingency tables. In addition, the Ω-shrinkage measure, which has shown the most conservative detection tendency in previous studies, was used [15]. These two metrics were employed with distinct analytical purposes. The Ω-shrinkage measure was used to mitigate statistical instability because of data sparsity, while the adjusted reporting odds ratio (aROR) was calculated to account for measured covariates available in the database. However, neither method can fully account for unmeasured confounding factors.

#### Disproportionality analysis

Reporting odds ratios (RORs) and 95% CIs were estimated using Eqs. (1) and (2) as follows:

where **a** represents targeted drugs with nephrolithiasis and urolithiasis, **b** represents non-targeted drugs with nephrolithiasis and urolithiasis, **c** represents targeted drugs without nephrolithiasis and urolithiasis, and **d** represents non-targeted drugs without nephrolithiasis and urolithiasis.

1 $${\rm{ROR}} = {{a/c} \over {b/d}}$$

2 $$95{\rm{\% CI}} = {\rm{exp}}\left\{ {\log \left( {{\rm{ROR}}} \right) \pm 1.96\sqrt {{1 \over a} + {1 \over b} + {1 \over c} + {1 \over d}} } \right\}$$

For the ROR, the criteria for defining positive signals for drug-drug interactions were adopted based on previous studies [16–18]. These criteria included (1) the lower limit of the 95% confidence interval of the ROR for the vitamin D combination group exceeding 1.0, (2) the ROR for the vitamin D combination group being higher than that in the other index groups (carbonic anhydrase inhibitors and vitamin D alone), and mutually exclusive 95% confidence intervals. We analyzed the impact of vitamin D preparations on nephrolithiasis and urolithiasis associated with carbonic anhydrase inhibitors using RORs and 95% CIs. We performed multivariate logistic regression analysis by including sex, age, and year of reporting as covariates to determine the aROR. Sex and age were selected as covariates because they are known physiological risk factors for urolithiasis. The reporting year was included to adjust for temporal reporting bias. The *p*-values were calculated using the Wald test derived from the logistic regression model.

#### Ω shrinkage measure

The Ω shrinkage measure prepares the 4 × 2 contingency table (Supplementary Fig. S1) when targeted drugs are used in combination, when each targeted drug is used alone, and when all other drugs are used, and the signal is obtained by dividing the observed value by the expected value. The method of calculating the signal is shown in detail in Eq. 3.

3 $${\rm{\Omega }} = {\rm{log}}2{{n111 + 0.5} \over {E111 + 0.5}}$$

n111: the reported number of adverse events associated with a targeted two-drug combination.

E111: the expected number of adverse events associated with a targeted two-drug combination.

4 $${\rm{\Omega }}025{\rm{ }} = \Omega - {{\Phi \left( {0.975} \right)} \over {{\rm{log}}\left( 2 \right)\sqrt {n111} }}$$

where ϕ(0.975) was 97.5% of the standard normal distribution and Ω025 > 0 was used as the threshold to screen for signals associated with a two-drug combination.

All statistical analyses were performed using JMP® Student 18.2.0 software (SAS Institute Inc., Cary, NC, USA). Statistical significance was set at *p* < 0.05. Categorical data were summarized as numbers (%).

#### Sensitivity analyses

To assess the consistency of the results and address potential confounders by indication (e.g., osteoporosis), we performed a sensitivity analysis using bisphosphonates (BP), which are osteoporosis treatments without carbonic anhydrase inhibitory activity, to rule out confounding factors associated with the underlying disease.

### Research involving human participants

The JADER dataset used in this study contained anonymized processed information in accordance with Japan’s Personal Information Protection Law. Furthermore, according to the ethical guidelines for clinical research in Japan, studies utilizing anonymized processed information do not require review by an ethical review committee.

## Results

### Study population

**Fig. 1:**
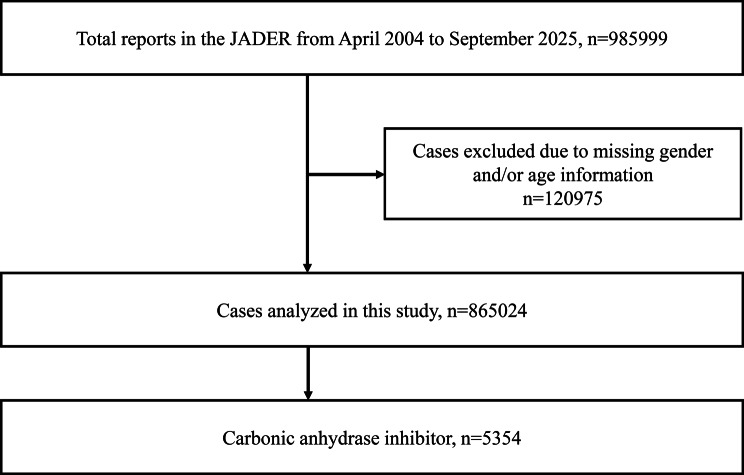
Selection flowchart of the study population. In total, 985,999 cases were identified in the JADER database. After excluding 120,975 cases, the remaining cases (*n* = 865,024) were analyzed. AE: adverse event. n: the number of reports (e.g. n+++: the number of all reports, n111: the number of drug D1 and drug D2 induced target AE reports)

A flow chart summarizing the process of selecting the study population is shown in Fig. 1. The dataset included 985,999 reports from April 2004 to September 2025. We excluded 120,975 reports with incomplete data and used the remaining 865,024 reports for further analysis.

Table 2 shows the characteristics of patients who used carbonic anhydrase inhibitors. In the carbonic anhydrase inhibitor group, 2768 (51.7%) patients were men. Approximately 25% were elderly (≥70 years old). 61 cases of renal and urinary tract stone disease were reported in patients taking carbonic anhydrase inhibitors.

**Table 2:** Background of study population using carbonic anhydrase inhibitors

	Carbonic anhydrase inhibitor, n (%)
Sex	
Male	2768 (51.7%)
Female	2586 (48.3%)
Age, years	
0–9	606 (11.3%)
10–19	543 (10.1%)
20–29	457 (8.5%)
30–39	483 (9.0%)
40–49	483 (9.0%)
50–59	550 (10.3%)
60–69	853 (15.9%)
70–79	891 (16.6%)
80–89	435 (8.1%)
90–99	51 (1.0%)
>100	2 (0.0%)
Renal and urinary tract stone disease	61

Supplementary Table S1 shows the drugs and their associated classifications related to reported cases of renal and urinary stones. Topiramate and zonisamide accounted for the majority of the reported carbonic anhydrase inhibitors.

### Signal detection for urinary tract stones

Table 3 shows the results of the disproportionality analysis for nephrolithiasis and urolithiasis associated with carbonic anhydrase inhibitors. When carbonic anhydrase inhibitors were combined with vitamin D preparations, a significant positive signal was observed for renal and urinary tract stone disease exclusively (aROR 59.85; 95% CI 29.04–123.36, *p* < 0.001), compared to that with carbonic anhydrase inhibitor (aROR 12.99; 95% CI 9.69–17.41; *p* < 0.001) or vitamin D alone (aROR 8.68, 95% CI 6.98–10.79, *p* < 0.001). A significant positive signal was also detected for the Ω shrinkage measure (Ω025 = 0.429).

**Table 3:** Disproportionality analysis and Ω shrinkage measure of renal and urinary tract stone disease associated with carbonic anhydrase inhibitors (CAI) and vitamin D (VD) preparations

	Renal and urinary tract stone disease	Without Renal and urinary tract stone disease	n111/E111	Ω/Ω025	Crude ROR	95% CI	Adjusted ROR	95% CI	*p*-value
CAI	53	5107	―	―	17.83	13.41–23.71	12.99	9.69–17.41	<0.001
VD	105	25686	―	―	7.02	5.69–8.68	8.68	6.98–10.79	<0.001
CAI＋VD	8	186	8/2.657	1.429/0.429	73.91	36.22–150.82	59.85	29.04–123.36	<0.001
Non-target drugs	485	833394	―	―	1.00	―	1.00	―	―

ROR, reporting odds ratio; CI: confidence interval

In the sensitivity analysis for BP, no cases of renal or urinary stones were reported in the group receiving concomitant CAIs and BP, whereas a significant signal persisted in the CAI and vitamin D groups (Supplementary Table S2).

## Discussion

In this study, our analysis of the JADER database revealed a significant positive signal for renal and urinary tract stone disease associated with the drug-drug interaction of carbonic anhydrase inhibitors and vitamin D preparations. Notably, the primary purpose of this disequilibrium analysis is not to establish a definitive causal relationship but to detect signals and generate hypotheses. However, the fact that a significant interaction signal was detected not only in the disproportionality analysis using ROR but also in the Ω shrinkage measure, which has shown a conservative detection tendency in previous studies [15], supports the plausibility of the hypothesis.

To improve the reliability of signal detection, we used two distinct metrics: the Ω shrinkage measure to mitigate statistical instability because of data sparsity and the adjusted ROR (aROR) to account for measured covariates. The consistency of results across these statistical methodologies suggests that the detected signal was relatively stable within the exploratory analysis. Regarding the effect of covariate adjustment, the crude ROR for the combination of CAI and VD was 73.91. However, after adjusting for sex, age, and reporting year, the aROR decreased slightly to 59.85. In general, the incidence of urinary stones is higher in men and is said to increase with age [19, 20]. This decrease suggests that demographic factors, such as older age in the combination group, contributed in part to the crude ROR estimate. However, the signal remained extremely high, even after adjustment. This suggests that the observed signal cannot be explained solely by demographic confounding factors and that specific pharmacological interactions between these drugs may contribute. The results of our sensitivity analysis using bisphosphonates lend additional support to this finding. Analysis using bisphosphonates detected no signal, suggesting that the observed signal was specific to the combination with vitamin D and was not solely because of confounding by the underlying disease (osteoporosis).

Vitamin D preparations have been reported to promote calcium absorption in the gastrointestinal tract and may cause hypercalciuria [21, 22]. This raises concerns that the combined use of these medications in patients taking CAIs may further increase the risk of kidney stone formation due to the synergistic effect of increasing urinary calcium excretion.

The risk of developing urinary stones can be reduced by altering the diet, lifestyle, and fluid intake [23–26]. Further, Considering the many types of antiepileptic drugs and osteoporosis medications available, and considering measures, such as changing the medication, which can be taken to avoid these risks, our findings may help prevent the onset of urinary tract stones in patients with epilepsy and osteoporosis. The number of patients suffering from urinary stones is increasing annually [27, 28], and stones have been indicated as a risk factor for chronic kidney disease, fractures, and cardiovascular events [29, 30]. Patients with epilepsy are noted to be at a high risk of future cardiovascular events, and this risk may be further increased by the presence of urinary tract stones [31]. Further, as the presence of urinary stones increases the risk of future urinary tract infections and post-renal kidney damage [30, 32], their prevention is extremely important.

Patients with epilepsy are at a high risk of fracture [8, 9]. Given that patients with epilepsy are prone to vitamin D deficiency [10], large-scale studies based on real-world data are needed to confirm the hypothesis presented in this study and to determine whether the combined use of these drugs increases the risk of developing urinary stones.

Several limitations should be considered when interpreting these results. First, the database consists of spontaneous reports; therefore, it is difficult to avoid reporting biases such as underreporting, notoriety bias, ripple effect, and Weber effect. Second, as the JADER database does not provide the actual number of cases in which each drug was used, the absolute risk of an event is difficult to determine. Third, as data on laboratory tests (e.g., serum calcium levels) were lacking, the risk of renal and urinary stones, including serum calcium levels and acid-base balance could not be determined. Fourth, some cases had to be excluded because of missing data. Fifth, the causal relationship between renal and urinary stones and medications could not be fully evaluated. Sixth, insufficient data was available for detailed dosage, and whether the risk increased in a dose-dependent manner. Seventh, we were unable to determine the stone composition (e.g., whether they were calcium phosphate stones). Eighth, the small number of events resulted in wide 95% confidence intervals, suggesting the results are unstable and that the reporting odds ratio (ROR) may be prone to overestimation. Furthermore, this limited event count precluded adjustment for other potential confounding factors (e.g., thiazides, renal function, underlying diseases). Additionally, because of the small sample size, we evaluated CAIs and vitamin D preparations as broad therapeutic classes, rather than evaluating individual drug-specific characteristics. Although we conducted a multivariate logistic regression, limitations regarding covariate adjustment in the disproportionality analysis must be acknowledged. As noted by Noguchi et al. [33], spontaneous reporting systems often lack complete covariate information. Important potential confounders, such as renal function, exact treatment duration, and comprehensive concomitant medications, were not available and thus could not be controlled; therefore, the estimated aRORs may still be subject to residual confounding. Finally, the CAIs analyzed in this study were predominantly zonisamide and topiramate, with a limited number of acetazolamide cases. As zonisamide and topiramate possess multiple pharmacological mechanisms beyond carbonic anhydrase inhibition (e.g., sodium channel blockade), determining from this database whether the observed risk is attributable solely to CA inhibition, is difficult. Previous studies have reported that drug-induced stone formation is primarily associated with metabolic acidosis and hypocitraturia because of CA activity, suggesting that our results likely reflect this mechanism.

## Conclusion

Our findings present the hypothesis that the drug-drug interaction between carbonic anhydrase inhibitors and vitamin D preparations may increase the incidence of renal and urinary stones. Therefore, in patients using carbonic anhydrase inhibitors, our findings suggest that selecting appropriate osteoporosis medications may be necessary to manage the risk of urinary tract stones. However, further pharmacoepidemiological studies using large-scale claims databases or electronic health records are needed to assess the impact, particularly the risks, associated with the concurrent use of carbonic anhydrase inhibitors and vitamin D preparations.

## Electronic supplementary material

Below is the link to the electronic supplementary material.

Supplementary material 1

Supplementary Material 2

Supplementary Material 3: : **Supplementary Figure S1**: Four-by-two contingency table for the evaluation of drug-drug interaction. AE: adverse event. n: the number of reports (e.g. n+++: the number of all reports, n111: the number of drug D1 and drug D2 induced target AE reports).

## Data Availability

The data used in this study are open data and can be obtained from the PMDA website: https://www.pmda.go.jp/safety/info-services/drugs/adr-info/suspected-adr/0004.html

## Abbreviations

JADER: The Japanese Adverse Drug Event Report
PT: Preferred Term
RORs: Reporting odds ratios
CI: Confidence interval
aRORs: adjusted Reporting odds ratios
