# Supplementary material for: Drug-drug interaction signals between carbonic anhydrase inhibitors and vitamin D preparations in urinary tract stones: disproportionality analysis evaluation from Japanese spontaneous reports of adverse events
Source: J Pharm Health Care Sci. 2026 Apr 27;12:56. doi: 10.1186/s40780-026-00574-2 (PMC13262390; doi:10.1186/s40780-026-00574-2)
Supplement: Supplementary file 1 — Supplementary material 1 [file 40780_2026_574_MOESM1_ESM.docx]

**Table S1: Drugs associated with reports of Renal and urinary tract stones and drug-related classification**

|  | Renal and urinary tract stone disease | Drug-related classification  (S/C/I) | CAI | VD |
| --- | --- | --- | --- | --- |
| CAI | 53 | 47/6/0 | Acetazolamide: 5  Zonisamide: 34  Topiramate: 14 |  |
| VD | 105 | 68/32/5 |  | Tacalcitol: 1  Calcitriol: 2  Alfacalcidol: 42  Eldecalcitol: 60 |
| CAI＋VD | 8 | CAI: 6/2/0  VD: 3/5/0 | Zonisamide: 5  Topiramate: 3 | Alfacalcidol: 6  Eldecalcitol: 2 |

S: Suspected drugs

C: Concomitant drugs

I: Interacting drugs
