# Supplementary material for: Drug-drug interaction signals between carbonic anhydrase inhibitors and vitamin D preparations in urinary tract stones: disproportionality analysis evaluation from Japanese spontaneous reports of adverse events
Source: J Pharm Health Care Sci. 2026 Apr 27;12:56. doi: 10.1186/s40780-026-00574-2 (PMC13262390; doi:10.1186/s40780-026-00574-2)
Supplement: Supplementary file 2 — Supplementary Material 2 [file 40780_2026_574_MOESM2_ESM.docx]

**Table S2.** Disproportionality analysis and Ω shrinkage measure of renal and urinary tract stone disease associated with carbonic anhydrase inhibitors (CAI) , vitamin D (VD) preparations and bisphosphonates (BP)

|  | Renal and urinary tract stone disease | Without Renal and urinary tract stone disease | n111/E111 | Ω/Ω025 | Crude ROR | 95% CI | Adjusted ROR | 95% CI | p-value |
| --- | --- | --- | --- | --- | --- | --- | --- | --- | --- |
| Non-target drugs | 471 | 811805 | ― | ― | 1.00 | ― | 1.00 | ― | ― |
| CAI | 53 | 5038 | ― | ― | 18.13 | 13.63–24.12 | 13.33 | 9.92–17.89 | < 0.001 |
| VD | 85 | 19622 | ― | ― | 7.47 | 5.92–9.41 | 8.92 | 7.04–11.31 | < 0.001 |
| BP | 14 | 21589 | ― | ― | 1.12 | 0.66–1.90 | 1.51 | 0.88–2.59 | 0.131 |
| CAI+VD | 5 | 147 | 8/2.657 | 1.429/0.429 | 58.63 | 23.93–143.60 | 47.94 | 19.62–118.92 | < 0.001 |
| CAI+BP | 0 | 69 | 3/1.289 | 0.968/-0.664 | ― | ― | ― | ― | ― |
| VD+BP | 20 | 6064 | ― | ― | 5.69 | 3.63–8.90 | 8.05 | 5.10–12.73 | < 0.001 |
| CAI+VD+BP | 3 | 39 | ― | ― | 132.58 | 40.83–430.51 | 113.35 | 33.90–379.02 | < 0.001 |

ROR, reporting odds ratio

CI: confidence interval

※The medications studied included bisphosphonates (etidronic acid, pamidronic acid, alendronic acid, ibandronic acid, risedronic acid, zoledronic acid, minodronic acid).
