# Supplementary material for: Drug-drug interaction signals between carbonic anhydrase inhibitors and vitamin D preparations in urinary tract stones: disproportionality analysis evaluation from Japanese spontaneous reports of adverse events
Source: J Pharm Health Care Sci. 2026 Apr 27;12:56. doi: 10.1186/s40780-026-00574-2 (PMC13262390; doi:10.1186/s40780-026-00574-2)
Supplement: Supplementary file 3 — Supplementary Material 3: : Supplementary Figure S1: Four-by-two contingency table for the evaluation of drug-drug interaction. AE: adverse event. n: the number of reports (e.g. n+++: the number of all reports, n111: the number of drug D1 and drug D2 induced target AE reports). [file 40780_2026_574_MOESM3_ESM.pptx]

## Slide 1
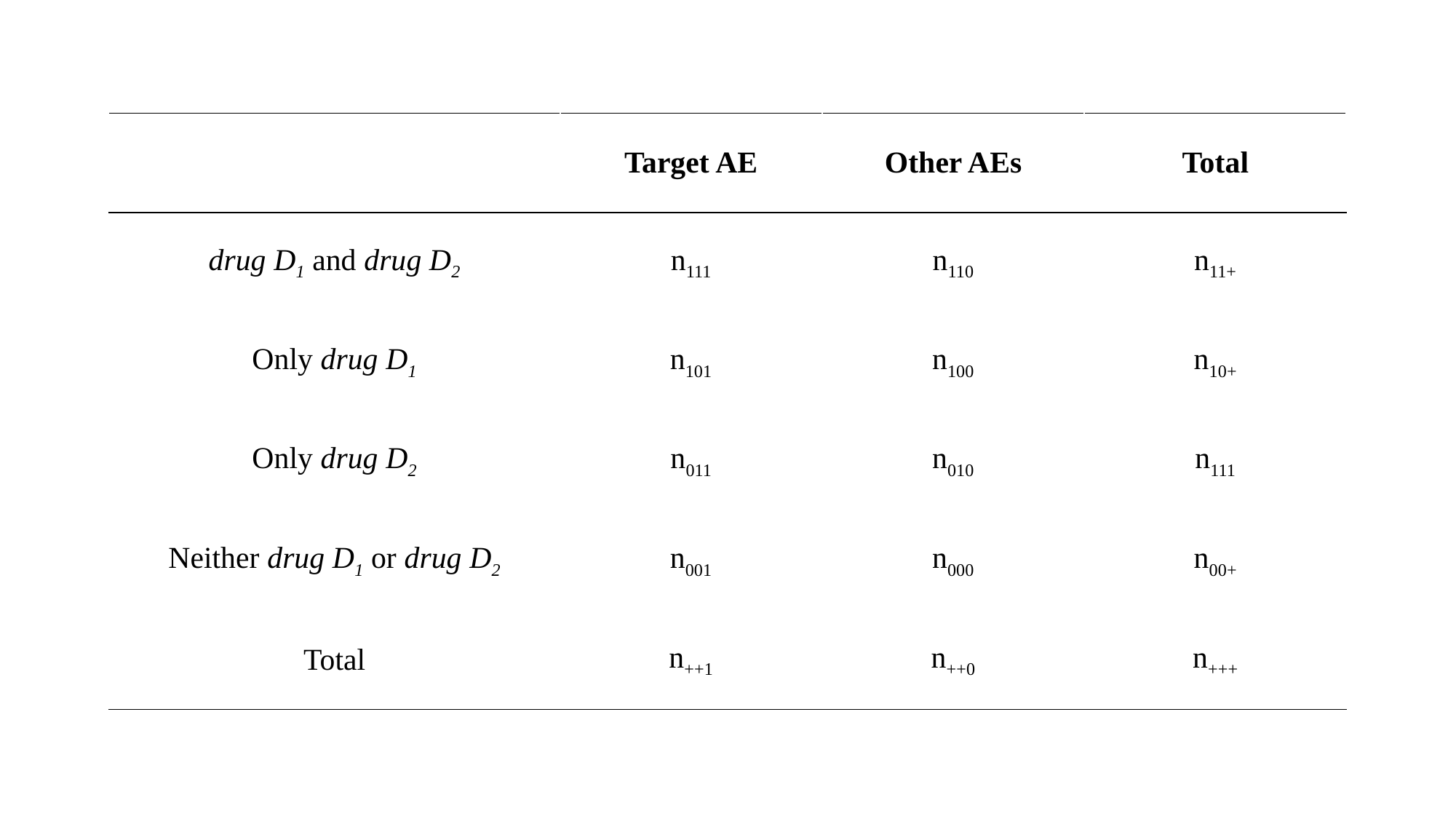

| | Target AE | Other AEs | Total |
| --- | --- | --- | --- |
| drug D1 and drug D2 | n111 | n110 | n11+ |
| Only drug D1 | n101 | n100 | n10+ |
| Only drug D2 | n011 | n010 | n111 |
| Neither drug D1 or drug D2 | n001 | n000 | n00+ |
| Total | n++1 | n++0 | n+++ |
